# The post-pandemic era: will physical distancing be a perceived way of life?

**DOI:** 10.12688/f1000research.52779.2

**Published:** 2022-01-13

**Authors:** Soban Qadir Khan, Jehan Al-Humaid, Faraz Ahmed Farooqi, Mohammed M. Gad, Muhanad Al-Hareky, Faisal Al-Onaizan, Fahad A. Al-Harbi

**Affiliations:** 1 Department of Dental Education, College of Dentistry, Imam Abdulrahman Bin Faisal University, Dammam, Saudi Arabia; 2 Department of Preventive Dental Sciences, College of Dentistry, Imam Abdulrahman Bin Faisal University, Dammam, Saudi Arabia; 3 Department of Substitutive Dental Sciences, College of Dentistry, Imam Abdulrahman Bin Faisal University, Dammam, Saudi Arabia; 4 Department of Restorative Dental Sciences, College of Dentistry, Imam Abdulrahman Bin Faisal University, Dammam, Saudi Arabia

**Keywords:** COVID-19, Post-pandemic, Lifestyle, physical distancing, Coronavirus, Saudi Arabia

## Abstract

**Background:** This study aimed to determine whether people living in the Eastern Region of Saudi Arabia would prefer to continue the practice of physical distancing after the coronavirus disease 2019 (COVID-19) pandemic or to return to their previous way of life.

**Methods: **This cross-sectional study was conducted from August 2020 to October 2020 in the Eastern Region of Saudi Arabia. A pre-tested questionnaire was sent electronically through social media. Data on participants’ demographics and their perspectives regarding post-pandemic physical distancing were collected. The calculated sample size was 1,066; however, the total number of responses included in the analysis was 989.

**Results:** The average age of the participants was 31.15±11.93 years. There were 435 men and 554 women in the study. Participants showed significantly high levels of disagreement with statements indicating that they were willing to use public transportation (61%), attend social gatherings (36%), and hug relatives or colleagues (40%) after the pandemic ( *p*<0.001); however, 43% agreed that they would spend time with family or friends ( *p*<0.001). The level of education was also found to be significantly related to the responses, and the level of disagreement increased as the level of education increased ( *p*<0.001).

**Conclusions:** One-third of the study participants planned to continue engaging in physical distancing even after the current pandemic and if the COVID-19 related restriction will remain in place for longer, there is chance to increase in this proportion. However, it cannot be concluded whether or not this behavior will prevail in the long run, after the ease in restrictions. If so, it may greatly affect some businesses and perhaps some social norms and values as well.

## Introduction

The novel coronavirus (coronavirus disease 2019 [COVID-19]) belongs to the same family of viruses (coronaviruses) as the Middle East respiratory syndrome coronavirus (MERS-CoV) and severe acute respiratory syndrome coronavirus (SARS-CoV). ^1, 2^ Many respiratory viruses are believed to transmit over multiple routes, including droplets, aerosols, and fomites. ^4^ Respiratory droplets moving from one person to another and contact with contaminated surfaces and objects are the primary sources of transmission. ^5, 6^ Presymptomatic transmission is the second type of transmission, in which the virus is transmitted from an infected person who has yet to develop symptoms to another person. ^7^

Various guidelines have been issued to reduce the spread of the pandemic, including avoiding handshaking or any type of physical contact, avoiding social gatherings or visiting family or friends, wearing masks and gloves, closure of public venues, and tourism and travel restrictions. ^2^ Increasing handwashing, minimizing face touching, wearing masks in public, and physical distancing are the measures that have been adopted globally. ^1^ Because COVID-19 returns in waves, the psychological impacts of physical distancing will persist over time and may indeed become accentuated with repeated iterations of physical distancing. Given that physical distancing affects the types of activities in which one can engage and impacts how activities are carried out, it is likely that this accounts for some of the psychological impact. ^8^

The effectiveness of physical distancing is determined by individual behavior. ^9^ The interventions most essential for control of pandemics necessarily disrupt social processes. Public measures were implemented in response to COVID-19; people were encouraged by authorities, media, and peers to voluntarily adopt “personal distancing” behaviors to reduce virus transmission (e.g., avoiding physical contact or close proximity with non-household members and reducing use of shared public spaces). ^10^

The present study population consisted of people living in Saudi Arabia, a Middle Eastern country, and Saudi society can be classified as a “high-context culture.” ^11^ Saudi people rely heavily on nonverbal communication, such as kissing and hugging as a greeting or welcome and as a sign of respect. ^12^ In contrast, physical distancing measures can feel unnatural ^11, 13^; however, regular practice of certain behaviors over a long period of time can make them automatic or habitual. ^14^ In addition, people are becoming comfortable with limiting socialization and following precautionary measures due to the secondary reinforcement gained as a consequence. ^13, 15^ To the best of our knowledge, the literature has yet to consider the possible post-pandemic-era lifestyles and behaviors of the people living in the Eastern Region of Saudi Arabia. Therefore, this study aimed to determine whether people living in the Eastern Region of Saudi Arabia would prefer to continue the practice of physical distancing after the coronavirus disease pandemic or to return to their previous way of life.

## Methods

This cross-sectional study was conducted in the Eastern Region of Saudi Arabia. The study was conducted from August 2020 to October of 2020. Institutional approval was obtained from the College of Dentistry, Imam Abdulrahman Bin Faisal University (approval number 202162).

The inclusion criteria were as follows: (1) aged between 18 and 70 years, and (2) residing in the Eastern Region of Saudi Arabia. The exclusion criterion was not being able read and understand English or Arabic. To calculate the required sample size Raosoft software (Seattle, Washington, USA) was used with, response distribution was set at 50%, the margin of error was set at 3%, and the confidence interval was set at 95%. Total population in Eastern region of Saudi Arabia, was obtained through general authority of statistics, Saudi Arabia. ^16^ Hence, the calculated sample size was 1066. However, to collect the required number of responses, convenient sample collection technique was used.

The questionnaire for the study included questions related to demographics and physical distancing. Precautions and instructions related to physical distancing provided by the World Health Organization and United States Centers for Disease Control, and these precautions were used to develop the items of the questionnaire. Initially, the questionnaire was written in English and translated into Arabic. The English version of the questionnaire was translated into Arabic by an Arabic language expert who was also proficient in English. Then the Arabic version was translated back into English by another expert in both languages. After developing the questionnaire, a pilot study was performed and 40 responses were collected, among the Saudi population living in Eastern region, to test the validity of the questionnaire, and the kappa statistic was 0.79. Hence, the revision of the questionnaire was not required.

In line with COVID-19 precautions, the questionnaire was administered electronically. Questionpro software (Dallas, TX, USA) was used for preparing the survey online. The questionnaire contained a total of 16 questions; however, questions regarding personal identification were not included to keep the responses anonymous. Responses for each item were based on a 5-point Likert scale (1 = “Never,” 2 = “Seldom,” 3 = “Sometimes,” 4 = “Frequently,” 5 = “Always”). To simplify the analysis and presentation of results, the response categories (1 to 5) were grouped into three categories: disagree (after combining never and seldom), neutral (sometimes), and agree (after combining frequently and always). All authors of the study then sent the survey link to all their WhatsApp contacts. The authors also requested from their contacts to share the links further.

The data were collected in Excel and later transferred to and coded in SPSS version 23 (IBM Corp., Armonk, NY, USA) for analysis. Frequency distributions and bar diagrams were constructed for descriptive analysis and presentation of the data. Normality of the data was tested first using the Shapiro-Wilk test, and the results were nonsignificant, indicating that the data were normally distributed. Hence, parametric tests were used for inferential analysis. A one-sample chi-square test was used to analyze the significance of the proportion of responses for each question. A chi-square test was used to compare participants’ demographics with their responses to questions related to physical distancing. The level of significance was set at P < 0.05.

### Ethical approval

Ethical approval for the research was obtained from the Research Unit of College of Dentistry, Imam Abdulrahman Bin Faisal University. The ethical approval letter number was EA:202162.

### Consent

A summary of the study was presented at the start of the survey along with a consent statement. Consent was implied by the submission of the completed anonymous survey. Written consent was not appropriate, given the anonymous nature of the survey. Participants had to click on the “Next” button to proceed with the questions, which were all on the participants’ plans with regard to physical distancing behavior after the COVID-19 pandemic.

## Results

The current study included 989 participants who completed and submitted the survey. A total of 1,350 individuals started the survey, and 989 completed it, resulting in a response rate of 73.5%. The mean age of the participants was 31.15 ± 11.93 years, with a range of 20 to 61 years. The majority of the participants were Saudi (705 [70%] Saudi participants and 284 [30%] non-Saudi participants). There were 435 (44%) men and 554 women (56%). Most of the participants were undergraduate students 474 (48.1%), followed by graduated 217 (22%), postgraduates 143 (15%) and 150 (15%) were undertaking qualifications at high school level. Overall, 677 (68%) lived with an average of 4 to 6 adults and 2 children.

**Figure 1.**
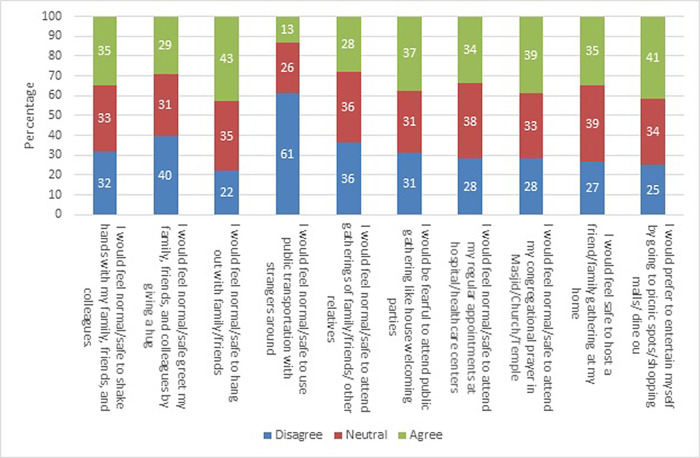
Participant perspectives regarding post-coronavirus disease 2019 (COVID-19) physical distancing behavior.

Figure 1 shows the responses to the post-COVID-19 survey of the entire study population. The percentage of disagreement was highest for the item on using public transportation with strangers, and the difference between disagree and agree was statistically significant (87% vs. 13%) (P < 0.001). Similarly, the percentage of disagreement was significantly higher for the items on attending gatherings of close family/friends/other relatives (72% vs. 28%) (P < 0.001) and greeting family, friends, and colleagues with a hug (71% vs. 29%) (P < 0.001). The highest percentage of agreement among all items was observed for spending time with family or friends (43%), which was significantly high (P < 0.001).

### Greetings involving physical contact (shaking hands and hugging)

This section of the questionnaire evaluated participants’ sense of safety in engaging in handshaking and hugging when greeting others after the pandemic ( Table 1). Non-Saudi residents were significantly higher in terms of disagreement with the items on handshaking (P < 0.0001) and hugging family or friends during greetings (P < 0.001). Women were less likely to disagree with the items on greeting by hugging than men (36% vs. 45%, P < 0.012). The level of education was found to be significantly associated with participants’ responses (P < 0.0001). There was no significant variation in responses between the size of family with handshaking, but the participants with 1-3 individuals in the home were significantly more likely to disagree with the item on hugging family or friends (P < 0.003).

**Table 1.** Participant demographic characteristics and sense of safety in engaging in physical greetings after COVID-19.

		I would feel normal/safe shaking hands with my family, friends, and colleagues			I would feel normal/safe greeting my family, friends, and colleagues with a hug		
		Disagree n(%)	Neutral n(%)	Agree N(%)	Disagree n(%)	Neutral n(%)	Agree n(%)
**Nationality**	**Saudi**	185(26.4)	242(34.5)	274(39.1)	244(35)	216(31)	240(34)
	**Non-Saudi**	125(44.6)	86(30.7)	69(24.6) *	146(52)	90(32)	43(15) *
**Gender**	**Men**	147(34)	129(30)	159(37)	194(45)	119(27)	122(28)
	**Women**	167(30)	200(36)	187(34)	198(36)	191(35)	163(30) *
**Education**	**Postgraduate**	56(39)	43(30)	44(31)	66(46)	42(30)	34(24)
	**Graduate**	96(44)	72(33)	49(23)	109(50)	70(32)	38(18)
	**Undergraduate**	121(26)	170(36)	182(38)	168(36)	155(33)	149(32)
	**High school or less**	39(26)	44(29)	67(45) *	48(32)	43(29)	59(39) *
**Family size**	**1-3**	108(35)	103(33)	98(32)	140(46)	92(30)	75(24)
	**4-6**	103(28)	131(36)	135(37)	129(35)	123(33)	117(32)
	**7-9**	54(29)	58(32)	72(39)	66(36)	50(27)	68(37)
	**Above 10**	49(39)	37(29)	41(32)	57(45)	45(35)	25(20) *

* Statistically significant at P < 0.05.

### Social activities (spending time with friends/family, hosting/attending gatherings, shopping)

Table 2 presents the participants’ responses with regard to their sense of safety in engaging in social activities after the pandemic. Saudi nationals were significantly more likely to agree with the items on spending time with family or friends (49% vs. 27%, P < 0.001), attending family gatherings (33% vs. 16%), hosting gatherings (43% vs. 15%, P < 0.001) and going for picnics or to shopping malls (48% vs. 25%, P < 0.001). Gender was not found to be associated with agreement with the items on engaging in social activities; however, women were significantly more fearful regarding attending public gatherings (P < 0.002). Level of education was found to be significantly associated with agreement with the items on spending time with family or friends, attending or hosting gatherings, and going out for self-enjoyment (P < 0.0001). Families with 4 to 6 people were significantly more likely to agree with the items on engaging in social activities like spending time with friends or family and attending parties (P = 0.001, 0.002 and 0.033, respectively).

**Table 2.** Participant demographic characteristics and their sense of safety in participating in social activities after coronavirus disease 2019 (COVID-19).

		I would feel normal/safe hanging out with family/friends			I would feel normal/safe attending gatherings of family/friends/other relatives			I would feel safe hosting a friends/family gathering at my home			I would be fearful to attend public gatherings like housewarming parties			I would prefer to entertain myself by going to picnic spots/shopping malls/dining out		
		Disagree n(%)	Neutral n(%)	Agree n(%)	Disagree n(%)	Neutral n(%)	Agree n(%)	Disagree n(%)	Neutral n(%)	Agree n(%)	Disagree n(%)	Neutral n(%)	Agree n(%)	Disagree n(%)	Neutral n(%)	Agree n(%)
**Nationality**	**Saudi**	121(17)	234(33)	344(49)	216(31)	256(37)	229(33)	141(20)	261(37)	299(43)	214(31)	222(32)	265(38)	132(19)	232(33)	337(48)
	**Non-Saudi**	98(35)	106(38)	76(27)*	139(50)	96(34)	45(16)*	120(43)	120(43)	41(15)*	96(34)	83(30)	101(36)	112(40)	97(35)	71(25)*
**Gender**	**Men**	105(24)	152(35)	176(41)	173(40)	149(34)	113(26)	125(29)	168(39)	143(33)	164(38)	118(27)	153(35)	124(28)	145(33)	167(38)
	**Women**	116(21)	193(35)	245(44)	186(34)	206(37)	162(29)	138(25)	216(39)	200(36)	147(27)	191(34)	216(39)*	123(22)	187(34)	243(44)
**Education**	**Postgraduate**	37(26)	60(42)	46(32)	65(45)	53(37)	25(17)	48(34)	65(45)	30(21)	41(29)	37(26)	65(45)	50(35)	45(31)	48(34)
	**Graduate**	71(33)	78(36)	68(31)	107(49)	70(32)	40(18)	86(40)	84(39)	47(22)	74(34)	66(30)	77(35)	71(33)	70(32)	75(35)
	**Undergraduate**	85(18)	157(33)	229(49)	148(31)	182(38)	143(30)	95(20)	186(39)	193(41)	132(28)	154(33)	187(40)	91(19)	170(36)	213(45)
	**High school or less**	28(19)	48(32)	74(49)*	39(26)	48(32)	63(42)*	34(23)	48(32)	68(45)*	60(40)	51(34)	39(26)*	34(23)	44(29)	72(48)*
**Family size**	**1-3**	80(26)	118(38)	110(36)	131(42)	104(34)	74(24)	93(30)	130(42)	86(28)	96(31)	98(32)	115(37)	88(28)	101(33)	120(39)
	**4-6**	65(18)	125(34)	179(49)	110(30)	153(41)	106(29)	81(22)	143(39)	146(39)	115(31)	126(34)	128(35)	81(22)	123(33)	165(45)
	**7-9**	42(23)	55(30)	87(47)	60(33)	61(33)	63(34)	41(22)	68(37)	75(41)	56(30)	56(30)	72(39)	35(19)	67(36)	82(45)
	**Above 10**	34(27)	47(37)	45(36)*	58(46)	37(29)	32(25)*	48(38)	43(34)	36(28)*	44(35)	29(23)	54(43)	43(34)	41(32)	43(34)

### Essential/routine activities (transportation, hospital checkups, prayers)

Nationality had a significant statistical association with agreement with the items on routine activities, such as using public transportation (P = 0.05), attending places of prayer (P = 0.017), and visiting the hospital for checkups or appointments (P = 0.0001) ( Table 3). Men were more likely to agree with the item on congregational prayers in religious venues (P = 0.0001). Participants with higher education were less likely to agree with the items on using public transportation (P = 0.013), visiting hospitals or healthcare places for regular checkups, and attending congregational prayer in masjids, churches, or temples (P = 0.005). It was also found that family size was not statistically associated with any of these essential/routine activities ( Table 3).

**Table 3.** Participants’ demographic characteristics and sense of safety in carrying out essential/routine activities after coronavirus disease 2019 (COVID-19).

		I would feel normal/safe using public transportation with strangers around			I would feel normal/safe attending my regular appointments at hospitals/healthcare centers			I would feel normal/safe attending my congregational prayer at the masjid/church/temple		
		Disagree n(%)	Neutral n(%)	Agree n(%)	Disagree n(%)	Neutral n(%)	Agree n(%)	Disagree n(%)	Neutral n(%)	Agree n(%)
**Nationality**	**Saudi**	407(58)	191(27)	103(15)	172(25)	261(37)	268(38)	181(26)	237(34)	283(40)
	**Non-Saudi**	193(69)	61(22)	26(9)*	102(36)	112(40)	67(24)*	98(35)	84(30)	99(35)*
**Gender**	**Men**	270(62)	109(25)	56(13)	120(28)	163(37)	153(35)	102(23)	121(28)	213(49)
	**Women**	336(61)	145(26)	73(13)	159(29)	213(38)	182(33)	180(32)	202(36)	172(31)*
**Education**	**Postgraduate**	99(69)	24(17)	20(14)	47(33)	60(42)	36(25)	39(27)	48(34)	56(39)
	**Graduate**	147(68)	52(24)	18(8)	75(35)	81(37)	61(28)	86(40)	59(27)	72(33)
	**Undergraduate**	270(57)	139(29)	64(14)	117(25)	187(39)	170(36)	114(24)	169(36)	191(40)
	**High school or less**	87(58)	38(25)	25(17)*	39(26)	47(31)	64(43)*	42(28)	47(31)	61(41)*
**Family size**	**1-3**	196(63)	75(24)	38(12)	96(31)	126(41)	87(28)	88(28)	112(36)	109(35)
	**4-6**	218(59)	101(27)	50(14)	103(28)	140(38)	127(34)	100(27)	127(34)	143(39)
	**7-9**	108(59)	50(27)	26(14)	43(23)	68(37)	73(40)	48(26)	49(27)	87(47)
	**Above 10**	84(66)	28(22)	15(12)	37(29)	42(33)	48(38)	46(36)	35(28)	46(36)

## Discussion

After the outbreak of the current pandemic, physical distancing was proposed and implemented as a primary method of reducing its spread. Recommended physical distancing measures can be divided into three categories: (1) avoid contact with others (e.g., handshakes and hugs), (2) avoid using or visiting publicly shared places, and (3) avoid participating in religious and non-religious gatherings. Studies evaluating the changes in lifestyle due to restrictions imposed as precautionary measures have been conducted across the globe. ^17, 18^ However, the present study was conducted to evaluate the perceived post-pandemic lifestyle.

In the analysis of the questions related to physical distancing behaviors. With regard to public transportation, only 13% agreed that they would use public transportation even after the pandemic. This cautious behavior will have a significant impact on the transportation industry. The industry must develop strategies to reduce this impact and plan well for the post-pandemic era. Hao *et al*., for example, have proposed a disaster management framework and post-pandemic agenda for the hotel industry. ^19^ In the present study, over 70% of participants also showed their disagreement or neutrality when asked about attending gatherings of family and friends, or hugging them. The nature of these three parameters are relevant to many aspects of life after COVID-19; people will be in closed environments and will have more contact. Similarly, as aerosols are known to be the primary method of spreading the virus, people will avoid hugging others. ^20^ Another notable aspect of the study findings was that about one-third of the respondents were unsure about practicing physical distancing after COVID-19.

Participants who were Saudi citizens showed more flexible behavior than non-Saudis, and it was found that there was a significantly higher percentage of Saudi citizens who reported wanting to return to their normal ways of life. For almost all questions being asked, the percentage of individuals who wanted to behave as they did before the pandemic was higher among Saudis compared with non-Saudis. These findings may be due to high context non-verbal communication among Arabs ^12^; the other groups of study participants were mostly non-Arabs. Hence, a significant difference in perspectives was observed.

The purpose of vaccines is to boost the human immune system in order for the body to better fight viruses. This is also true for COVID-19 vaccines. ^21^ Hence, the fear of getting infected or having any COVID-19 positive individual contact will remain even after the pandemic ends. ^22^ Therefore, the minimum distances that have been maintained between an infected person and a potential should be established based on scientific evidence. ^23^ In the present study, educated people are more aware of the virus and its effects even after vaccination or the end of the pandemic. So, those who were more educated indicated that they were more likely to continue practicing physical distancing after the pandemic.

The present study had some limitations. Data were only collected from the Eastern Region of Saudi Arabia. Hence, the study’s findings cannot be generalized to the entire population of the country. Similarly, nationality was just noted as Saudi or non-Saudi, and future studies should examine the specific nationalities of the participants. This would facilitate the analysis and country-wise or region-wise comparison of the data. Data collection was done through sending the survey link to personal contacts hence perhaps all sections of society might not be covered. Finally, the data collection process was completed before the development of the vaccine, after getting vaccinated people may feel safer and their responses may be different.

The overall impression from the study findings was that practicing physical activities and adopting a normal lifestyle after the pandemic will vary due to cultural and social norms and values. Societies that more commonly use non-verbal communication methods will be more likely to stop practicing physical distancing; however, education level was found to be negatively associated, and hence more educated people perhaps adopt physical distancing even after this pandemic.

Therefore, it can be concluded that engagement in physical activities and the carrying out of normal life after this pandemic will vary due to cultural and social norms and values. It was also observed that people will be either hesitant or avoid closed environments and public transportation. Future studies are recommended to evaluate the change in perception of post-pandemic physical distancing after the development of vaccines. In addition, if this perceived lifestyle remains for a long period, it will affect social life, and will have significant consequences on the culture and society.

## Data availability

### Underlying data

Harvard Dataverse: Post-Pandemic era: will physical distancing be a perceived way of lifestyle? https://doi.org/10.7910/DVN/IGSGU3 . ^24^

The project contains the following underlying data:

• Final Data Edidted.tab (The data file containing the complete data, which includes demographic variables and responses related to physical distancing behavior).

### Extended data

Harvard Dataverse: Post-Pandemic era: will physical distancing be a perceived way of lifestyle? https://doi.org/10.7910/DVN/IGSGU3 . ^24^

This project contains the following extended data:

• QuestionPro-Survey-7657352-PDF-Export-05-19-2021-T043556.pdf (Questionnaire used for data collection in this study in both English and Arabic).

Data are available under the terms of the Creative Commons Zero “No rights reserved” data waiver (CC0 1.0 Public domain dedication).
