# Supplementary material for: The post-pandemic era: will physical distancing be a perceived way of life?
Source: F1000Res. 2022 Jan 13;10:1090. Originally published 2021 Oct 27. [Version 2] doi: 10.12688/f1000research.52779.2 (PMC8796005; doi:10.12688/f1000research.52779.2)
Supplement: Peer review history

# Peer review: Reviewer response for version 2

**DOI:** 10.5256/f1000research.112731.r119617

**Published:** 2022-01-27

**Authors:** Nazeer Khan

**Affiliations:** 1 Director of Office of Research, Innovation and Commercialization, Shifa Tameer-e-Millat University, Karachi, Pakistan

The authors of the article, titled ' The post-pandemic era: will physical distancing be a perceived way of life?' have incorporated all the suggestions and corrected the sampling design and conclusion. Therefore, it is now ready to be indexed.

Is the work clearly and accurately presented and does it cite the current literature?

Partly

If applicable, is the statistical analysis and its interpretation appropriate?

Yes

Are all the source data underlying the results available to ensure full reproducibility?

No

Is the study design appropriate and is the work technically sound?

Yes

Are the conclusions drawn adequately supported by the results?

Partly

Are sufficient details of methods and analysis provided to allow replication by others?

Partly

Reviewer Expertise:

NA

I confirm that I have read this submission and believe that I have an appropriate level of expertise to confirm that it is of an acceptable scientific standard.

# Peer review: Reviewer response for version 2

**DOI:** 10.5256/f1000research.112731.r119616

**Published:** 2022-01-21

**Authors:** Dr. Shamta Sufia

**Affiliations:** 1 College of Public Health and Health Informatics, King Saud Bin Abdulaziz University for Health Sciences, Riyadh, Saudi Arabia

Changes adequately addressed.

Is the work clearly and accurately presented and does it cite the current literature?

Yes

If applicable, is the statistical analysis and its interpretation appropriate?

Yes

Are all the source data underlying the results available to ensure full reproducibility?

Yes

Is the study design appropriate and is the work technically sound?

Yes

Are the conclusions drawn adequately supported by the results?

Yes

Are sufficient details of methods and analysis provided to allow replication by others?

Yes

Reviewer Expertise:

dental public health

I confirm that I have read this submission and believe that I have an appropriate level of expertise to confirm that it is of an acceptable scientific standard.

# Peer review: Reviewer response for version 1

**DOI:** 10.5256/f1000research.56099.r99540

**Published:** 2021-12-09

**Authors:** Dr. Shamta Sufia

**Affiliations:** 1 College of Public Health and Health Informatics, King Saud Bin Abdulaziz University for Health Sciences, Riyadh, Saudi Arabia

The concept of identifying the opinions of people regarding physical distancing post-pandemic is interesting. However, for example in the Introduction some changes in the sequence of ideas can improve the flow e.g. the guidelines for avoiding infections (paragraph 3) can be presented before the effectiveness of social distancing can be addressed (paragraph 2) only after this taking up of recurrent waves of Covid infection seems logical. The transition to non verbal communication is quite abrupt and need reconsideration.

**Methods**

- On how many subjects was the questionnaire pilot tested and where was the questionnaire pilot tested?
- Were any changes made to the questionnaire?
- Did the sample cover responses from all sections of the society since personal contacts were used through social media.

**Results**

- For a non native English speaker the narrative becomes a little confusing as to what is meant by agreement/dis-agreement to the statements. A simple explanation for the reader would be more appropriate.

**Discussion**

- Using simple terms as individuals feeling of sense of security or otherwise would make reading less confusing.
- Discussing the limitation of the study the authors need to state the large sample size as a strength of the study, However, limiting it to one geographical region is a limitation. They also fail to identify religious factors that could have acted unfavorably on the study.

Some of the closing statements in the discussion are repetitive and hence can be rephrased.

Is the work clearly and accurately presented and does it cite the current literature?

Yes

If applicable, is the statistical analysis and its interpretation appropriate?

Yes

Are all the source data underlying the results available to ensure full reproducibility?

Yes

Is the study design appropriate and is the work technically sound?

Yes

Are the conclusions drawn adequately supported by the results?

Yes

Are sufficient details of methods and analysis provided to allow replication by others?

Yes

Reviewer Expertise:

dental public health

I confirm that I have read this submission and believe that I have an appropriate level of expertise to confirm that it is of an acceptable scientific standard, however I have significant reservations, as outlined above.

# Response

**Published:** 2021-12-16

**Authors:** soban khan

**Affiliations:** Imam abdulrahman bin Faisal University, Saudi Arabia

**Comment 1:** *The concept of identifying the opinions of people regarding physical distancing post-pandemic is interesting. However, for example in the Introduction some changes in the sequence of ideas can improve the flow e.g. the guidelines for avoiding infections (paragraph 3) can be presented before the effectiveness of social distancing can be addressed (paragraph 2) only after this taking up of recurrent waves of Covid infection seems logical. The transition to non verbal communication is quite abrupt and need reconsideration.*

**Reply:** Revised as suggested.

**Comment 2:** *On how many subjects was the questionnaire pilot tested and where was the questionnaire pilot tested?*

**Reply:** Revised and added in third para of method section.

**Comment 3:** *Were any changes made to the questionnaire?*

**Reply:** Not required because of having acceptable value kappa statistics.

**Comment 4:** *Did the sample cover responses from all sections of the society since personal contacts were used through social media.*

**Reply:** Added in the study limitations in the revised version.

**Comment 5:** *For a non native English speaker the narrative becomes a little confusing as to what is meant by agreement/dis-agreement to the statements. A simple explanation for the reader would be more appropriate.*

**Reply:** It is mentioned in the method that original questionnaire had 5-point Likert scale (1 = “Never,” 2 = “Seldom,” 3 = “Sometimes,” 4 = “Frequently,” 5 = “Always”). For the simplicity of the analysis and results presentation we merged them into 3 categories which was also mentioned in the fourth para of method section.

**Comment 6:** *Using simple terms as individuals feeling of sense of security or otherwise would make reading less confusing.*

**Reply:** Discussion has been revised.

**Comment 7:** *Discussing the limitation of the study the authors need to state the large sample size as a strength of the study, However, limiting it to one geographical region is a limitation. They also fail to identify religious factors that could have acted unfavorably on the study.*

**Reply:** Study limitations has been revised.

**Comment 8:** *Some of the closing statements in the discussion are repetitive and hence can be rephrased.*

**Reply:** Conclusion has been revised.

# Peer review: Reviewer response for version 1

**DOI:** 10.5256/f1000research.56099.r98081

**Published:** 2021-11-17

**Authors:** Nazeer Khan, Sajida Naseem, Sana Mangrio

**Affiliations:** 1 Shifa College of Medicine, Shifa Tameer-e-Millat University, Islamabad, Pakistan; 2 Director of Office of Research, Innovation and Commercialization, Shifa Tameer-e-Millat University, Karachi, Pakistan

**Is the work clearly and accurately presented and does it cite the current literature?**

 The article overall is written well, using appropriate study design and cites current literature, but some changes are required regarding methodology and conclusion.  

****

**Are sufficient details of methods and analysis provided to allow replication by others?**

 The sample size details are not mentioned in detail, how the sample 1066 was obtained, which prevalence was used in the Raosoft. What was the total population from which the representative sample was taken?  

****

 Simple random sampling technique and sampling frame not mentioned in detail about how the technique was employed. 

 The data collection does not align with the sampling technique. The sampling technique mentioned is Simple Random but data collection procedure states that a link was sent to WhatsApp contacts of the authors, which implies it is a convenient sampling method.  

****

**Are the conclusions drawn adequately supported by the results?**

 The conclusion in the abstract states that since 1/3 of participants planned to continue physical distance, it indicates that life will not be the same after the pandemic. The 1/3 result mentioned here is not the correct ratio to draw such conclusions.

Is the work clearly and accurately presented and does it cite the current literature?

Partly

If applicable, is the statistical analysis and its interpretation appropriate?

Yes

Are all the source data underlying the results available to ensure full reproducibility?

No

Is the study design appropriate and is the work technically sound?

Yes

Are the conclusions drawn adequately supported by the results?

Partly

Are sufficient details of methods and analysis provided to allow replication by others?

Partly

Reviewer Expertise:

NA

We confirm that we have read this submission and believe that we have an appropriate level of expertise to confirm that it is of an acceptable scientific standard, however we have significant reservations, as outlined above.

# Response

**Published:** 2021-12-16

**Authors:** soban khan

**Affiliations:** Imam abdulrahman bin Faisal University, Saudi Arabia

**Comment 1:** *The sample size details are not mentioned in detail, how the sample 1066 was obtained, which prevalence was used in the Raosoft. What was the total population from which the representative sample was taken? * 

**Reply:** It was not a prevalence study (like caries, diabetes, heart disease, etc) hence prevalence could not be obtained from literature. This study was conducted on general population and none of the specific group of the population was targeted hence for these kinds of scenarios 50% is generally used (which was used and mentioned). Secondly, regarding the total population of the Eastern region, we have added the text and reference in the method section.  

**Comment 2:** *Simple random sampling technique and sampling frame not mentioned in detail about how the technique was employed.*

**Reply:** Revised and sample frame is mentioned now in the revised version

**Comment 3:** *The data collection does not align with the sampling technique. The sampling technique mentioned is Simple Random but data collection procedure states that a link was sent to WhatsApp contacts of the authors, which implies it is a convenient sampling method. * 

**Reply:** Some revisions have been made in the respective parts.

**Comment 4:** *The conclusion in the abstract states that since 1/3 of participants planned to continue physical distance, it indicates that life will not be the same after the pandemic. The 1/3 result mentioned here is not the correct ratio to draw such conclusions.*

**Reply:** Revised in the new version.
